# The complete mitochondrial genome of *Hemerobius simulans* Walker (Neuroptera, Hemerobiidae)

**DOI:** 10.1080/23802359.2019.1675544

**Published:** 2019-10-11

**Authors:** Yuxin An, Cong Li, Xiaoying Li, Haoran Cui, Yuyu Wang

**Affiliations:** College of Plant Protection, Hebei Agricultural University, Baoding, PR China

**Keywords:** *Hemerobius simulans*, mitochondrial genome, phylogenetic relationships, Hemerobiidae

## Abstract

The complete mitochondrial (mt) genome of *Hemerobius simulans* Walker (Neuroptera, Hemerobiidae) is reported in this work. The whole mt genome is 17,985 bp long and contains 37canonical genes and an A + T-rich region, which is the same with insect ancestral mt genome arrangement. All 13 PCGs used the typical ATN as initiation codons. The control region of *H. simulans* mt genome is 1,416 bp long and the base composition is 90.0% of A + T. The phylogenetic analysis revealed that Hemerobiidae was monophyletic and was the sister group to Chrysopidae.

*Hemerobius simulans* Walker belongs to Hemerobiidae, which is the third largest group of Neuroptera and important natural enemies because both their adults and larvae prey on aphid, scale insect, worm eggs, and mollusc insects (Yang 1981). There are about 600 known species in the world and 130 species in China by 2016 (Zhao 2016). Mitochondrial (mt) genomes have been widely used in the study of taxonomy, phylogeny, evolution, population genetics and comparative genomics (Cameron 2014). However, the study of mt genomes from Hemerobiidae is still scarce. There are only two mt genomes (*Neuronema laminatum* and *Micromus angulatus*) from this family published on GenBank and there is no representative mt genome from Hemerobiinae reported.

Herein, we sequenced and annotated the mt genome of *H. simulans* Walker (GenBank accession number: MN315266) in this study. The speciemen used for DNA extraction was collected by Yunlan Jiang on 2018-10-6 at Wuling Mountain, Hebei Province, China (N40°33′50ʺ, E117°29′12ʺ). The voucher specimens (accession No.WYY003) are deposited in Hebei Agricultural University Museum. This mt genome, with a total length of 17,985 bp, contains 37 genes (13 PCGs, 22 tRNAs and 2 rRNA genes) and an A + T-rich region, which are typical in metazoan mt genomes (Wolstenholme 1992). The base composition of the whole mt genome is 39.5% of A, 39.6% of T, 11% of C, and 9.9% of G. There are 23 genes encoded by the main chain (J) and 14 genes encoded by the minority chain (N). There are 15 overlapping regions ranging from 1 to 8 bp and 12 intergenic regions ranging from 1 to 1835 bp in addition to the control region. The control region is 1,416 bp long and the base composition is 90.0% of A + T. All 13 PCGs use the typical ATN as initiation codon. For termination codon, 8 proteins use TAA; ND1 uses TAG; ND2, ND3, ND4, and ND5 use T-tRNA.

Phylogenetic trees were reconstructed with MrBayes 3.2.2 (Ronquist et al. 2012) and PhyML 3.0 online web server (http://www.atgc-montpellier.fr/phyml/) (Guindon et al. 2010) based on the concatenation data of 13 PCGs (only containing the first and second position) from 26 species of Neuroptera (Figure 1). Raphidioptera and Megaloptera were used as outgroups. Hemerobiidae was identified as monophyletic and being the sister group to Chrysopidae. Ascalaphidae was paraphyletic as to Myrmeleontidae. Psychopsidae was recovered as the sister group to the big branch including (Ithonidae, (Matispidae, (Hemerobiidae, Chrysopidae))), which need further studies to demonstrate.

**Figure 1.**
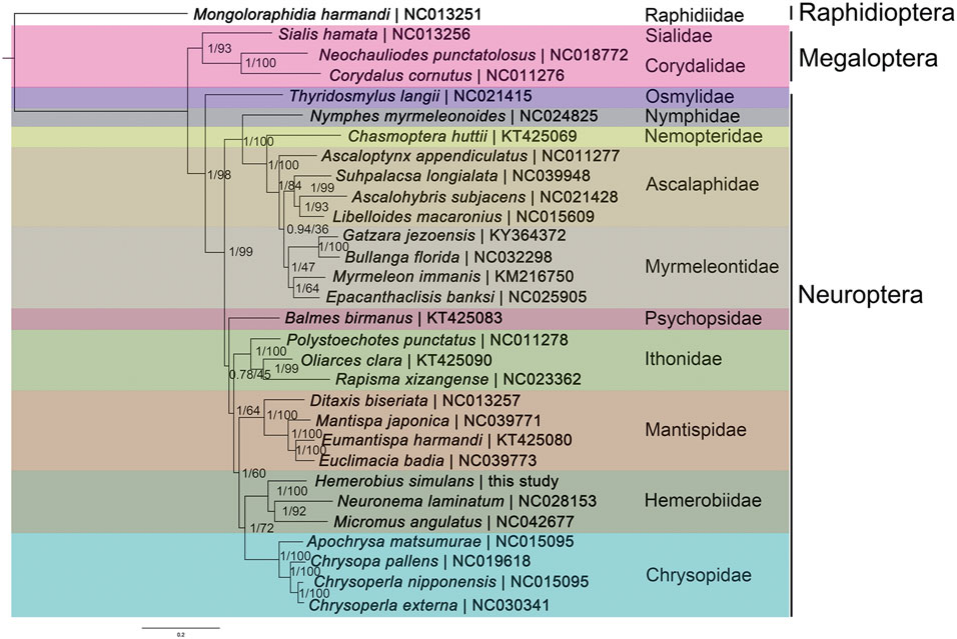
Phylogenetic trees inferred from BI and ML analysis based on the concatenation data of 13 PCGs (only containing the first and the second codon positions). The nodal numbers indicate the posterior probability (left) and the bootstrap support values (right). Genbank accession numbers for the sequences are indicated next to the species names.
